# Effect of Temperature and Strain Rate on the Thermomechanical Response and Dynamic Restoration Mechanisms of Ti-6Al-4V Alloy During Forging

**DOI:** 10.3390/ma19184004

**Published:** 2026-09-20

**Authors:** Shanli Salahi, Tolga Yılmaz, Onur Fevzi Kevenlik, Ganira Jabbarova, Vusala Musayeva, Ömer Asal, Hanifi Çinici

**Affiliations:** 1 Department of Metallurgical and Materials Engineering, Faculty of Technology, Gazi University, 06560 Ankara, Türkiye; tolgayilmaz@gazi.edu.tr (T.Y.); hcinici@gazi.edu.tr (H.Ç.); 2 Department of Defense Technologies, Institute of Science, Kırıkkale University, 71450 Kırıkkale, Türkiye; onurfevzi@icloud.com; 3 Department of Engineering and Applied Sciences, Azerbaijan State University of Economics (UNEC), AZ1001 Baku, Azerbaijan; ganira-jabbarova@unec.edu.az (G.J.); vusala.musayeva@unec.edu.az (V.M.); 4 Department of Manufacturing Engineering, Faculty of Technology, Gazi University, 06560 Ankara, Türkiye; omerasal@gazi.edu.tr

**Keywords:** Ti-6Al-4V alloy, hot deformation, thermomechanical processing, strain rate, microstructural evolution

## Abstract

The thermomechanical behavior of the Ti-6Al-4V (Ti64) alloy under hot deformation is determined by a complex interplay between strain hardening and thermally assisted restoration processes that finally lead to the formation of the resultant microstructure and mechanical properties. In this research, the effects of deformation temperature (700, 800, and 900 °C) and strain rates (low, moderate, and high) on microstructural evolution, hardness, and dislocation density of Ti64 alloy were systematically studied. Deformed samples were prepared by hot forging under specified deformation conditions, followed by characterization using optical microscopy, scanning electron microscopy (SEM), X-ray diffraction spectroscopy (XRD) analysis, and Vickers hardness (HV3) testing. The results demonstrated a gradual change from the stable α + β lamellar microstructure at 700 °C to fragmented lamellae and severe globularization at 900 °C, evidencing the increased significance of dynamic recovery (DRV), dynamic recrystallization (DRX), and globularization with increasing deformation temperature. The hardness distribution was strongly dependent on the deformation location, with the outer regions exhibiting higher hardness than the specimen centers; the maximum hardness of approximately 365 HV was obtained in the outer region at 900 °C under high deformation, whereas the center region remained comparatively softer. This spatial variation was attributed to the competition between deformation-induced dislocation accumulation and thermally activated softening. XRD analysis revealed that dislocation density greatly depends on a synergistic influence of deformation temperature and strain. The maximum dislocation density was achieved at moderate deformations at 700 °C and at high deformations at 800 °C, while a significant decrease took place at 900 °C under severe deformations, proving the dominance of thermally activated processes of recovery and recrystallization. Williamson–Hall analysis further indicated that lattice strain and microstrain did not always follow the same trend, confirming that macroscopic lattice distortion and local crystallographic strain represent distinct aspects of the deformation response. Thus, the comprehensive analysis of the microstructure, mechanical properties, and crystallographic features demonstrates that deformation temperature has a crucial influence on the strain hardening vs. dynamic softening balance. Among the investigated conditions, deformation at 900 °C with a relatively low strain rate provided the most favorable combination of lamellar fragmentation, globularization, dynamic restoration, and a comparatively homogeneous hardness response, indicating its potential for controlled thermomechanical processing of Ti64.

## 1. Introduction

Ti64 alloy, known as the “workhorse” of the titanium industry for its excellent strength-to-weight ratio, improved corrosion resistance, and biocompatibility, remains essential in the aerospace, medical, and chemical industries [1,2,3]. In the aerospace industry, it is a major material choice for gas turbine engines and aircraft construction. In the medical field, it is widely used in orthopedics and dentistry for the manufacture of implants due to its unique biomechanical characteristics and biocorrosion resistance [1,3,4,5]. However, the material exhibits limited formability at low temperatures due to its relatively high α-phase content with a hexagonal close-packed (hcp) crystal structure [1,6]. The alloy normally forms a coarser lamellar structure with a solidification texture when cast [7]. This is not desirable from an industrial processing point of view because only equiaxed structures are needed to achieve higher ductility and superplasticity [1,3,6].

To address microstructural deficiencies, thermomechanical processing (TMP), such as hot forging, hot rolling, and hot compressive deformation, has been employed to facilitate the transition from lamellar microstructures to fine equiaxed grains [1,3,8]. In this respect, hot pressing and isothermal compression have been reported to be crucial techniques in the study of the material’s flow stress behavior [1,2,4]. It is imperative at this stage not only because it contributes to the formation of the final product but also because it helps eliminate any porosity within the final product, making it resistant to fatigue and enhancing its mechanical properties [3,5]. Furthermore, TMP technology helps control the microstructure of the material, preventing any inhomogeneity in the microtexture that could lead to its failure [3,9,10,11].

Plastic deformation in titanium alloys is a complex, multi-stage process governed by the interplay of work hardening and dynamic softening [5]. During hot deformation, material behavior is primarily controlled by processing parameters such as the direction of deformation, temperature, and strain rate [3,10,12,13]. Recent compression studies on metastable Ti alloys have further demonstrated that deformation conditions can strongly influence strain localization, phase transformation, microstructural evolution, hardness, and the resulting mechanical response, highlighting the importance of coordinated control of deformation parameters during thermomechanical processing [13,14]. In the initial stage of plastic flow, work hardening dominates as dislocation multiplication leads to a sharp rise in flow stress [2,3,5]. As deformation progresses, thermally activated softening processes, such as DRV, DRX, and dynamic globularization, become more prominent [15,16,17,18]. The relatively high stacking fault energy (SFE) in titanium alloys favors dislocation rearrangement and elimination via climb and cross-slip, thereby promoting dislocation-induced softening and DRV [3,4,19,20]. DRX further contributes to softening by nucleating and developing strain-free grains. This mechanism can proceed either discontinuously, known as discontinuous dynamic recrystallization (DDRX) via grain boundary bulging, or continuously, known as continuous dynamic recrystallization (CDRX) via subgrain rotation and the formation of high-angle grain boundaries (HAGB) from low-angle grain boundaries (LAGB) [4,21,22,23]. Additionally, spheroidization of the α-phase can occur as the β-phase penetrates α-lamellae through shear bands or zones of strain accumulation [1,2,6,24,25,26,27].

This phase change is fundamental to determining optimal operating parameters and mapping industrial processing [2,3]. Despite extensive studies on high-temperature deformation above 1000 °C and low-temperature deformation below 600 °C, the warm-to-hot temperature range remains a vital domain for microstructural improvement [2,3]. In such an alloy, under conditions ranging from about 700 °C to 900 °C, the kinetics of globularization and the transition from DRV to DRX show strong dependence on the strain rate [1,28,29]. The hot-compression investigations of near-α titanium alloys have similarly demonstrated that deformation temperature and strain rate strongly influence flow behavior, lamellar fragmentation, and dynamic recrystallization, with higher temperatures generally promoting recrystallization and microstructural refinement [30]. Controlling these factors is crucial to prevent macro-instabilities, such as adiabatic shear bands and fracture [2,3,31]. Chong et al. analyzed the hot deformation of lamellar Ti64 in the temperature range of 700–950 °C, and the deformation was divided into regimes. They revealed that DRX of α-lamellae is the primary globularization process, whereas nanotwin formation is secondary, in contrast to boundary splitting and subdivision at higher temperatures [32]. Similarly, Bodunrin et al. analyzed the hot compression of wrought Ti64 alloy with a complex initial microstructure in the temperature range of 750–950 °C, where dynamic globularization of the α phase becomes the prevailing mechanism of flow softening, and found a stable process at low sub-transus temperatures such as 800 °C [33]. Simultaneously, Seshacharyulu et al. studied hot working of Ti64 of extra-low interstitial (ELI) grade with a Widmanstätten preform within a temperature range of 750 °C to 1100 °C, which demonstrated that although lamellar globularization is effectively achieved by the dynamic shear mechanism at temperatures of 850 °C to 950 °C at lower strain rates, high strain rate processing at temperatures less than 950 °C leads to extensive flow instability and the formation of shear bands [34]. Park et al. investigated the hot workability of commercial Ti64 alloy over the temperature range of 850–1000 °C, where flow curves at 850 °C display pronounced flow softening effects due mainly to dynamic spheroidization and recovery, along with the formation of localized shear bands at higher strain rates [35]. Moreover, Perumal et al. studied structural behavior at subtransus temperatures of 880 °C and 950 °C and proved that flow softening and steady-state effects were mainly due to the gradual reorientation of α-laths perpendicular to the direction of compression loading, rather than only the initial lath thickness [36]. Zhang et al. studied a multidirectional isothermal forging process starting at an initial temperature of 850 °C, where grain size reduction in the coarse lamellar structure was achieved through the joint contribution of continuous dynamic recrystallization (CDRX) and discontinuous dynamic recrystallization (DDRX), which dramatically improves tensile strength and ductility at both room temperature and 400 °C while shifting the failure mode from brittle quasi-cleavage to a fully ductile dimple fracture [37]. The current research study investigates the warm-to-hot deformation characteristics of bulk Ti64 using isothermal hot compression tests conducted in both X and Y axes at three temperatures of 700 °C, 800 °C, and 900 °C, over a range of strain rates from low to high, and a constant deformation extent of 10%, respectively. The study aims to evaluate the influence of multi-axial deformation on microstructural changes, determine the primary deformation modes, and develop appropriate processing conditions for this vital alloy during its warm-to-hot working regime.

## 2. Materials and Methods

### 2.1. Material and Deformation Process

The thermomechanical behavior of the Ti64 alloy was investigated using hot deformation of rectangular samples with dimensions of 10 × 15 mm. The samples were heated and hot-pressed using a Hidroliksan brand, 160-ton capacity single-acting hydraulic press equipped with an integrated heating oven at deformation temperatures of 700, 800, and 900 °C, with ram feed rates of 0.5, 2, and 10 mm/s, respectively. The total deformation extent was kept constant at 10% for all experimental conditions, while the deformation temperature and ram feed rate were systematically varied to evaluate their effects on the microstructural evolution and mechanical response of the alloy. For quantitative characterization of the deformation conditions, the nominal initial strain rate ($\dot{\varepsilon_{0}}$) was calculated from the applied ram feed rate and the initial sample height using Equation (1) [38].

(1) $$\dot{\varepsilon_{0}}=\;\frac{\nu }{\;h_{0}}$$

where $\dot{\varepsilon_{0}}$ is the nominal initial strain rate (s^−1^), ν is the ram feed rate (mm/s), and h_0_ is the initial sample height (mm). Therefore, the corresponding strain rates to the ram feed rates of 0.5, 2, and 10 mm/s are calculated as 0.033, 0.133, and 0.667 s^−1^, respectively.

The hot deformation was performed along both the X and Y axes to investigate the effect of multidirectional deformation on the microstructural evolution and mechanical response of the alloy. Prior to the deformation procedure, samples underwent thermal treatment in an oven at a rate of 10 °C/min. Upon reaching deformation temperatures of 700, 800, and 900 °C, the samples were held at each temperature for half an hour to ensure thermal equilibrium. Following the heat treatment and hot deformation procedure, the samples were cooled to room temperature. The preparation procedure of metallographic samples involved grinding using up to #2500 grit with SiC abrasive papers, followed by polishing. The chemical composition of the reference Ti64 sample was determined by Bruker Q4 Matrix Tasman Model spectroscopic analysis equipment, with the obtained results presented in Table 1 and found to be consistent with the certified composition of the alloy [39].

### 2.2. Characterization of Samples

In the produced samples, the subsequent characterization analyses were consistently performed on the cross-sectional regions, irrespective of the deformation axis of X and Y, to ensure a consistent basis for comparison among the different deformation conditions. Microstructural characterization was performed on an Olympus inverted microscope and a JEOL JSM 6060 LV scanning electron microscope operating at an acceleration voltage of 20.0 kV. The hardness of the fabricated samples was measured using a Qness Q30M hardness tester under a load of HV3 (3 kgf) with a dwell time of 10 s. Measurements were performed at three different regions from the exterior to the center of the sample, with five indentations (n = 5) performed at each region. Therefore, the hardness value for each region was calculated as the average of the three measurements. Chemical composition and intermetallic particle distribution within the Ti64 alloy were evaluated using a Bruker D8 Advance X-ray diffractometer equipped with Cu-Kα radiation. XRD patterns were collected over a 2θ range of 10–90° with a step size (scan sensitivity) of 0.01° and a scanning rate of 2°/min.

## 3. Results and Discussion

**Figure 1:**
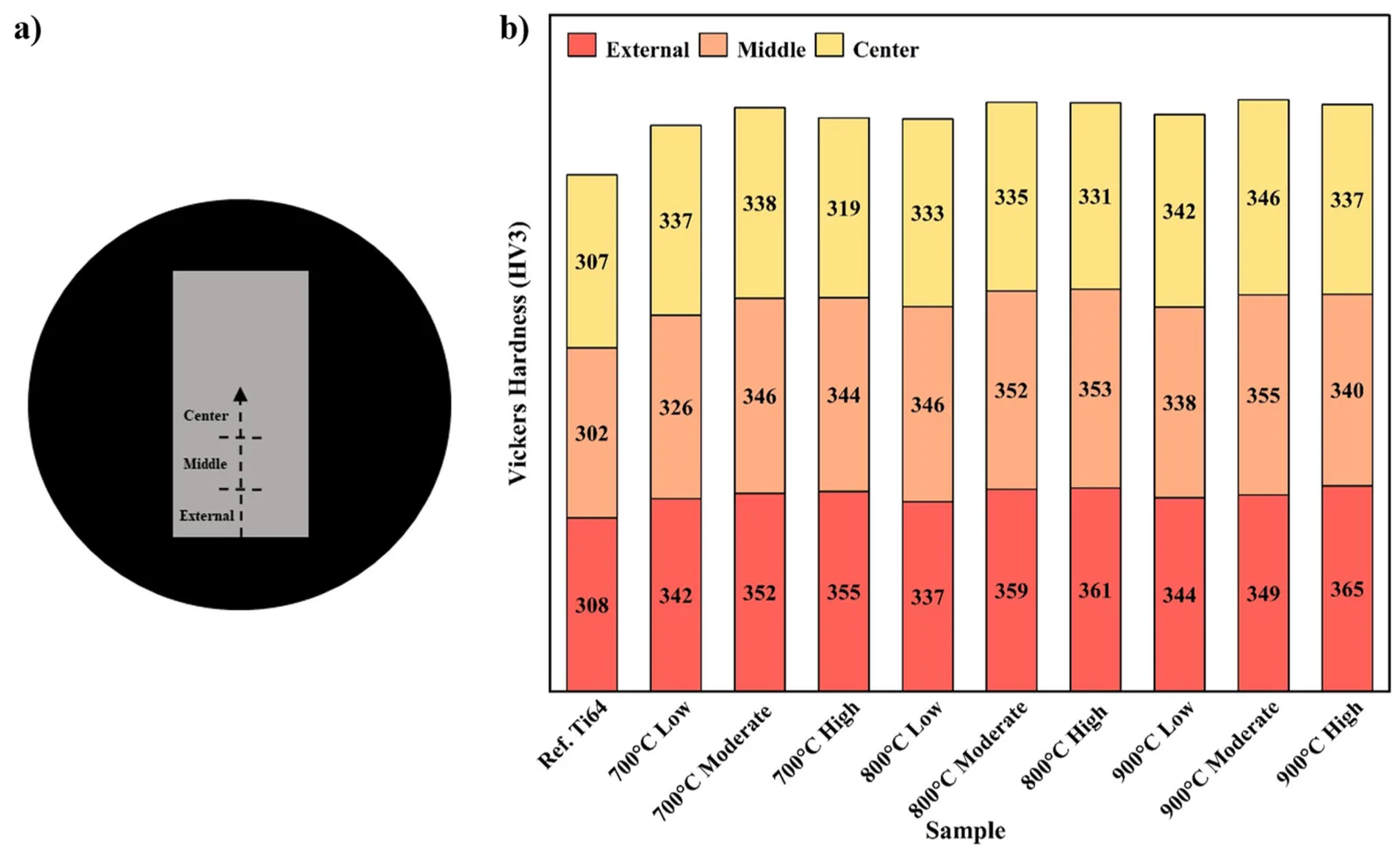
(**a**) Schematic illustrations of three different regions for Vickers hardness (HV3) measurements, (**b**) Vickers hardness (HV3) distribution of the fabricated samples.

The Vickers hardness (HV3) tests indicated that the deformation temperature and applied strain rate influenced the hardness profile of the samples, as shown in Figure 1.

At 700 °C, the outer-region hardness increased from 342 HV in the case of low deformation to 355 HV in the case of high deformation, while the center hardness decreased from 337 HV to 319 HV in the case of the highest level of deformation. Such behavior implies the presence of continuous strain hardening of the surface caused by dislocation multiplication, and intensive DRV of the center, causing the partial destruction and rearrangement of dislocations. Therefore, the process of deformation hardening dominated in the vicinity of the surface, while the thermally activated softening processes were more efficient in the interior of the sample. At 800 °C, the dependence between deformation and hardness was more consistent. The hardness of the outer region reached 361 HV, and the hardness of the middle region increased from 346 HV to 353 HV. At the same time, the hardness of the center remained almost constant between 331 HV and 335 HV. It is possible to conclude that the applied strain was sufficient to increase the hardness in the vicinity of the surface due to high levels of recovery connected with the elevated temperature of deformation. While in the external zone at 900 °C, the highest hardness of 365 HV is attained under high deformation, in the center zone, hardness values remain at a relatively low value of 337 HV. The increased temperatures tend to favor DRV and DRX of the grains; the high strain levels experienced by the grains near the surface have been sufficient to produce adequate dislocation accumulation and grain refinement, which counteract thermal softening. Conversely, the relatively low hardness values in the inner regions of the specimens indicate that DRV and DRX become increasingly effective where there is relatively low strain. According to the Hall-Petch equation, grain refinement leads to hardness improvement due to increased resistance of grain boundaries to dislocation movement [40,41,42].

**Figure 2:**
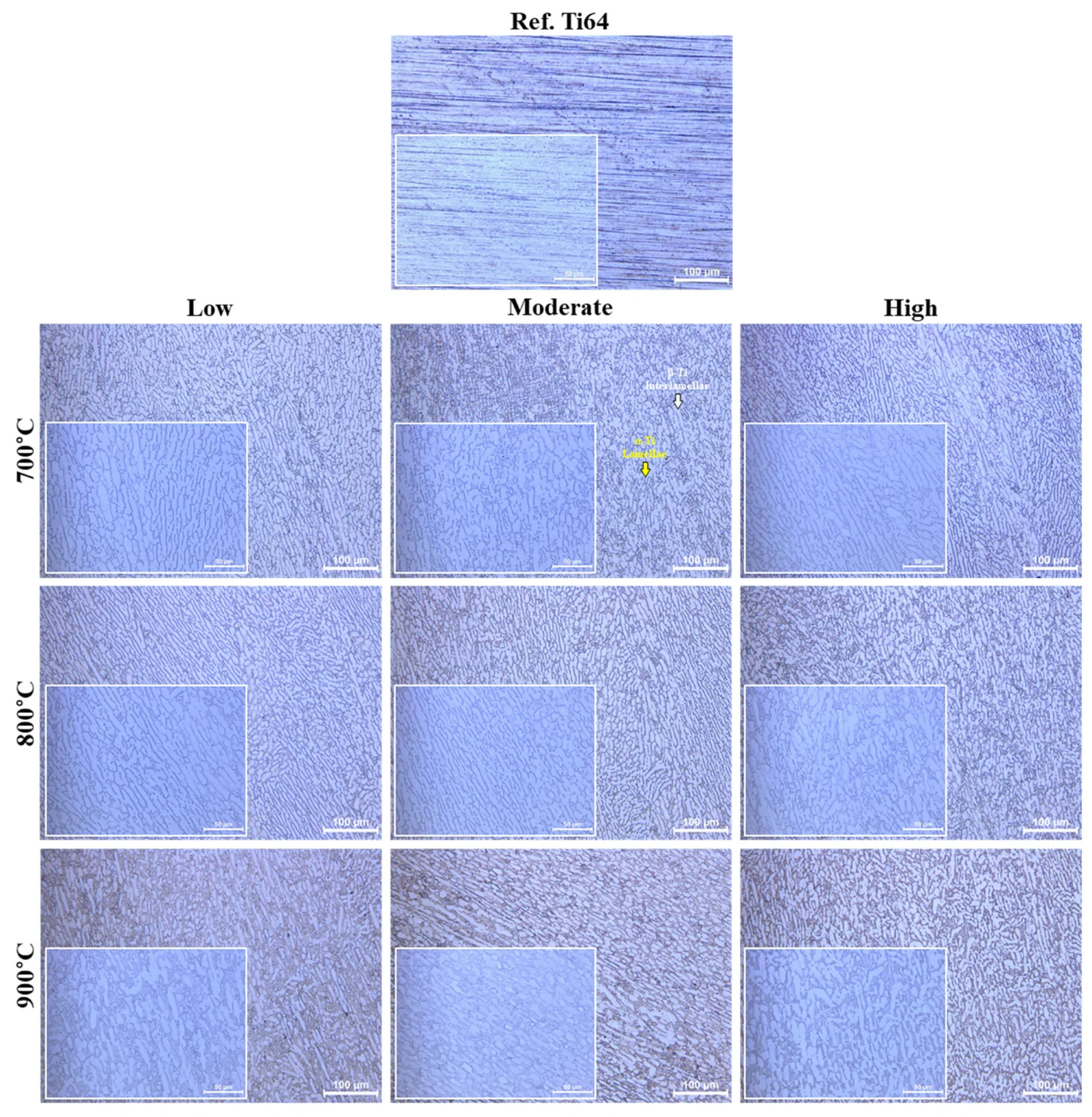
Optical microscopy observations of the microstructure of the Ti64 samples.

The structural analysis of the hot-pressed Ti64 alloy, as shown in the micrographs in Figure 2, indicates an intricate interplay between work hardening and thermally driven deformation softening over the temperature range 700 °C to 900 °C at different strain rates. The microstructure at the isotherm of 700 °C is characterized by the predominance of the lamellar structure at all applied strain rates. The results obtained are consistent with the restricted deformability of Ti64 at low temperatures, due to the significant volume fraction of the hcp α-phase, which restricts the number of active slip systems [3,6,25]. In such warm working conditions, DRV is used as the major restoring process, but sometimes it does not help completely to offset the high flow stress due to tangled dislocations and limited thermal activation [1,3,4,43,44,45].

The micrographs show that above a temperature of 800 °C, structural instability sets in due to localized bending and kinking of α lamella and β interlamellar regions. Kinking is recognized as a crucial step before globularization in a dynamic state. The process of kinking takes place due to rigid-body rotation of unfavorably oriented α lamella colonies for sliding, thus leading to the formation of shear bands [1,3,46,47,48]. Under low strain rate conditions, this temperature leads to better dislocation reorganization, causing jagged α/β interface structures, thus suggesting early stages of lamellae fragmentation, which is a diffusion-driven process where the β phase advances into the α lamellae to lower the total interface energy [1,6,49].

The 900 °C isotherm marks the clear boundary where the onset of hot working occurs, and thermal processes for softening, specifically DRX and dynamic globularization, become the dominant variables for the microstructure [2,50]. There is a clear pattern of lamellar break-up that can be observed, especially at lower strain rates, where the coarsely structured alloy becomes increasingly fine-grained [1,51,52]. The process involves dislocation recovery and cancelation in opposite directions along slip planes, thereby facilitating the formation of interface planes for β-phase infiltration and subsequent spheroidization [1,4,53]. On the other hand, at increased strain rates corresponding to 900 °C, the material exhibits elongation due to insufficient time for complete globularization, often resulting in flow localization rather than grain refinement [1,24,28,29].

**Figure 3:**
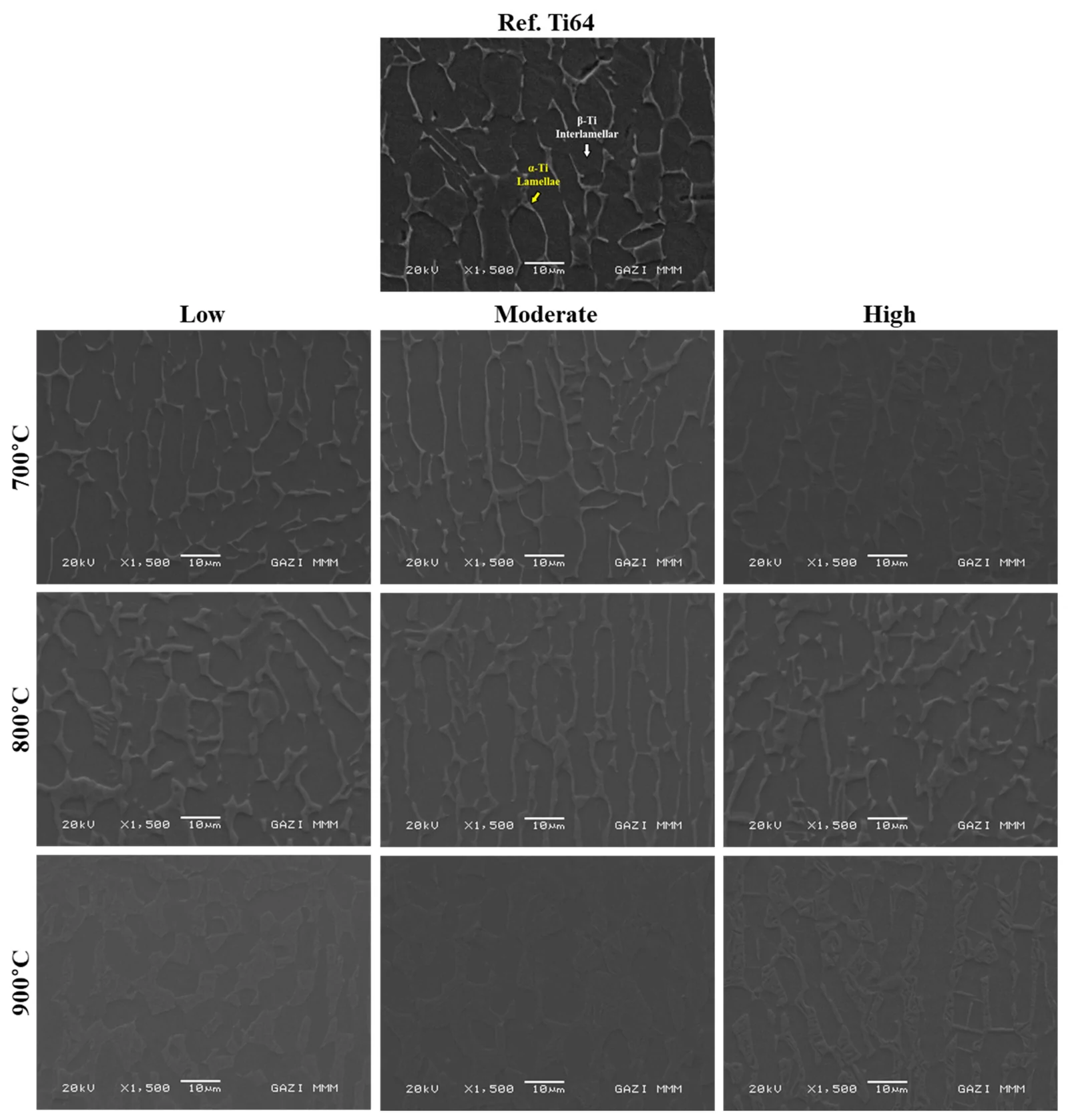
SEM micrographs for the microstructural morphology characterization of produced Ti64 samples.

The microstructural transformations of Ti64 at the sub-micron level through high-resolution SEM analysis, as shown in Figure 3, within the thermomechanical processing map, demonstrate the sensitivity of the α/β interfaces to changes in thermodynamic conditions.

The alloy preserves its rigid Widmanstätten structure at the 700 °C isothermal temperature, characterized by highly elongated α-Ti lamellae, with no signs of thermally induced restoration at any strain rate [1]. The stability of the alloy during warm deformation arises from restricted dislocation motion in the hcp α lattice, where work hardening and dislocation nucleation occur more often than DRX [1,6,25]. The alloy’s transition to the 800 °C isothermal condition signals a structural change, marked by bends in the lamellar colonies in the SEM micrographs [1]. This process is a necessary microstructural prelude to globularization, driven by colony misorientation relative to basal or prismatic slip, such that their orientation changes via rigid-body rotation, allowing them to meet the geometric requirements imposed by the compressive strain [1,46]. This is clearly demonstrated in backscattered electron (BSE) contrast in SEM images, highlighting orientation changes and the formation of shear bands that serve as potential nucleation sites for HAGBs [1,6].

The evolution of the system into the isothermal 900 °C region represents a change in the mechanism where diffusional mechanisms become dominant in controlling the refinement of microstructure [1]. In this regard, the formation of shorter equiaxed primary α-laths occurs at the low strain rate of the current isotherm through microscopic examination using SEM analysis [1]. The mechanism for the globular transformation is governed by the creation and annihilation of dislocations at shear planes that promote the nucleation of α/α interfaces to be invaded by the relatively ductile β phase to minimize interfacial energy [1,24,49]. On the other hand, SEM images taken at a temperature range of 900 °C under high strain rate loading show that the process of spheroidization is severely slowed down owing to the reduced time interval in which diffusion can take place [1,3]. Some level of fragmentation is seen, but overall the structure remains elongated and wavy, implying that flow localization and kinking continue to be the dominant deformation mechanisms even at high temperatures [1,3,31,49].

**Figure 4:**
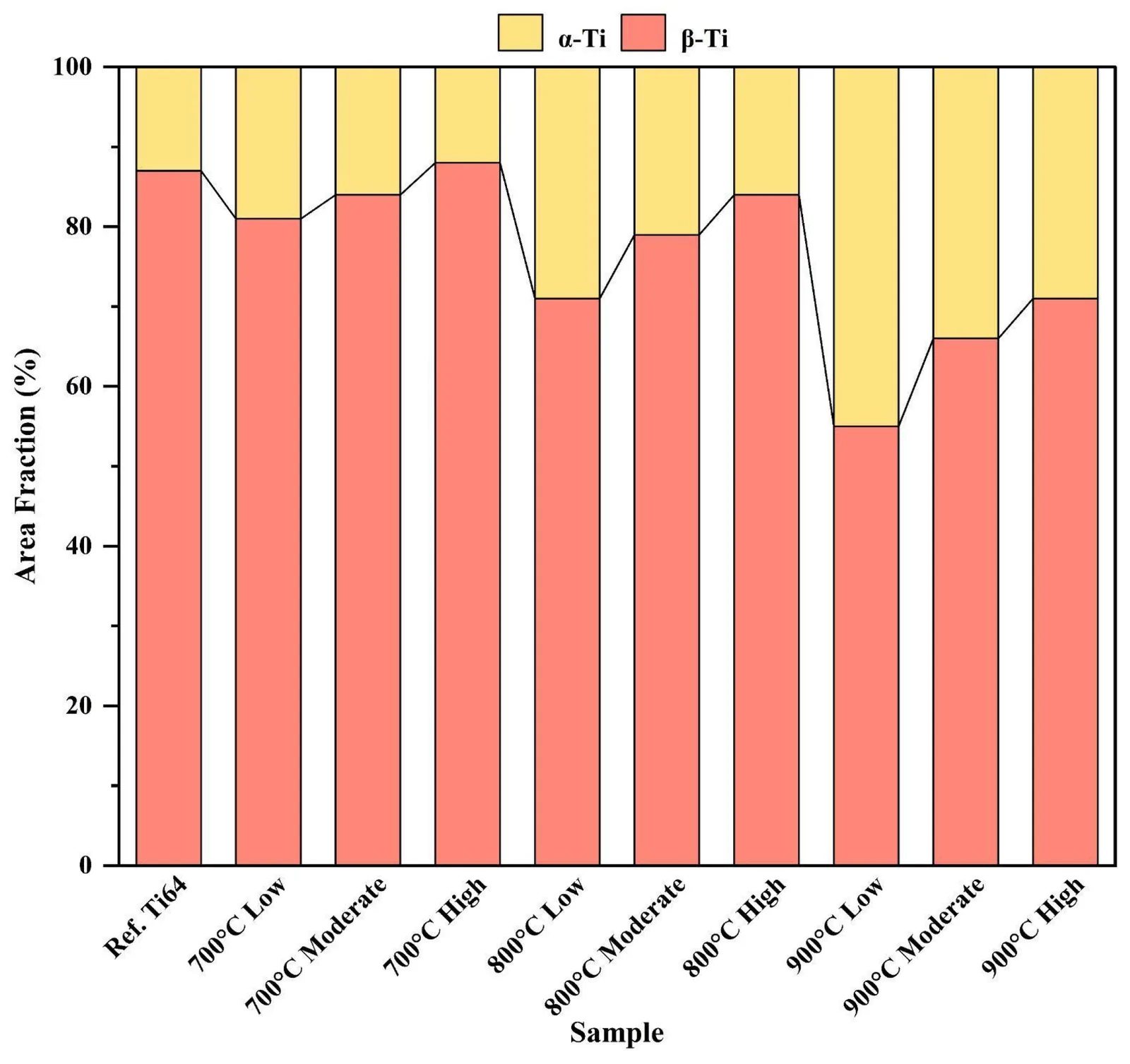
ImageJ-derived based quantification of the phase area fraction of samples.

The area fractions of the α and β phases were calculated from the SEM images using the ImageJ software 1.54d as shown in Figure 4 [54]. The obtained results demonstrated that the deformation temperature and strain rate had a considerable effect on the formation and distribution of the α and β phases during the hot deformation process. For the samples deformed at 700 °C, only slight variations in the measured phase area fractions were observed. The β-phase area fraction decreased from 87% in the reference sample to 81% under low levels of deformation, whereas the α-phase area fraction increased from 13% to 19%. By increasing the deformation, on the other hand, the β-phase area fraction slowly rose to 88%, whereas the α-phase area fraction decreased to 12%. Such relatively minor effects show that deformation at 700 °C was not enough to trigger globularization or any significant microstructural restructuring. Deformation was mainly due to accumulation of dislocations, bending, and kinking of the lamellae, and limited diffusion due to lower temperatures did not allow any substantial movement of interfaces and DRV. Such similar behavior of deformation in the α + β phase field has been reported by Ji et al. who reported that inadequate thermal activation inhibits microstructural evolution during hot deformation of Ti64 [55].

By raising the deformation temperature to 800 °C, even greater alterations of the phases were observed. Under low levels of deformation, the fraction of the β-phase decreased to 71%, and the α-phase fraction was 29%. With increasing deformation, the fraction of the β-phase increased up to 84%, and the fraction of the α-phase decreased to 16%. These observations indicate that higher temperatures of 800 °C contributed to lamellar fragmentation of the α colonies along with globularization. Higher temperature and hence faster diffusion make it easier for dislocations to be rearranged, for interfaces to migrate, and for boundaries to split, leading to transformation of the α + β structure. Microstructural development such as lamellar breakdown, globularization, and dynamic recrystallization in hot deformation is reported from studies conducted by Liu et al. [56]. Microstructural development was highly significant at the temperature of 900 °C, where the α-phase volume fraction attained 45% under low levels of deformation while the volume fraction of the β-phase reduced to 55%. With the increase in the levels of deformation, the volume fraction of the β phase increases to 71% while that of the α phase reduces to 29%. This indicates that deformation near the β-transus greatly improves the diffusion-controlled microstructural restoration process. At this temperature level, fragmentation of the lamellar colonies of the α phase, migration of the interfaces, dynamic globularization, and DRX become increasingly active. Callegari et al. showed that thermo-mechanical processing in the α + β range leads to globularization of the α lamellae along with the continuous transformation of the α/β structure [57]. As can be seen from the ImageJ analysis, the increase in the deformation temperature leads to an increasingly refined microstructure in Ti64. Despite the maintenance of the lamellar structure when deformed at 700 °C, lamellar fragmentation and globularization started to occur in samples subjected to deformation at 800 °C. In turn, DRV and DRX prevailed at 900 °C, leading to a more homogeneous α + β structure.

**Figure 5:**
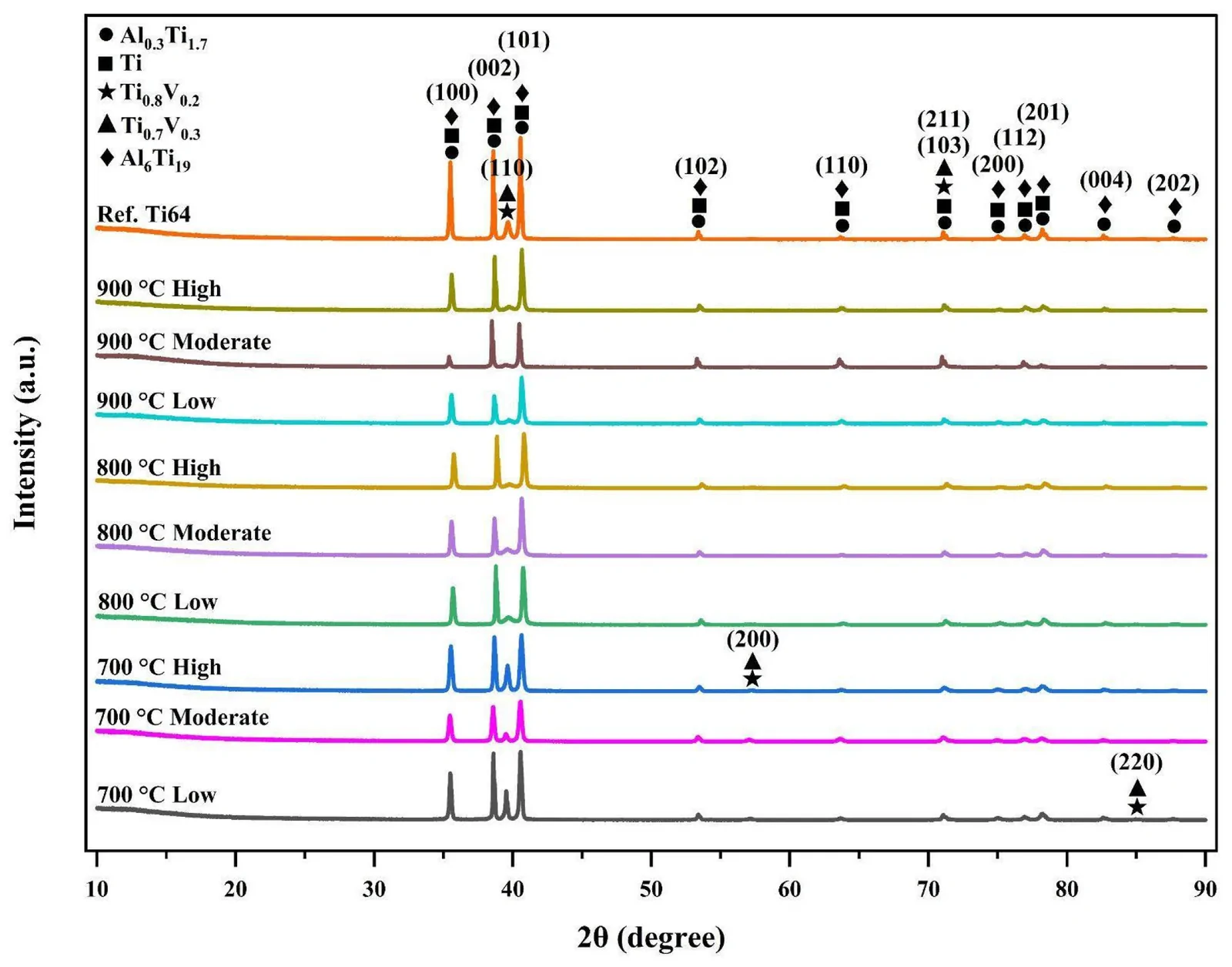
XRD patterns of the fabricated samples.

The phase composition and structural development of the Ti64 samples at different thermomechanical treatments were explained using the XRD technique, as shown in Figure 5. The diffraction data obtained for the hot forging samples between 700 °C and 900 °C under varied strain rates of high, medium, and low were compared with the Ref Ti64 to establish the primary phase of hcp α-Ti and body-centered cubic (bcc) β-Ti [58,59]. The Al_0.3_Ti_1.7_, Al_6_Ti_19_, Ti, Ti_0.8_V_0.2_, and Ti_0.7_V_0.3_ phases and precipitates were identified in the XRD analysis with ICDD card no. 01-077-6855, 03-065-8567, 00-001-1198, 01-081-9813, and 01-081-9817, respectively.

The reflections that can be observed at 2θ ≈ 35°, 38.5°, and 40.5° correspond to the crystallographic planes (100), (002), and (101), respectively, for the hcp phase of Al_0.3_Ti_1.7_ and Al_6_Ti_19_, characteristic of the α-Ti matrix. It can be observed from the changes in the (110) reflections around 39° to 40° that the effect of processing temperature and strain rates on the stability of the β-phase [58,59]. The area contains the cubic Ti_0.8_V_0.2_ and Ti_0.7_V_0.3_ solid solution phases that function as volume fraction markers of the β-Ti phase. Regarding Ti_0.7_V_0.3_, the primary (110) peak is identified at 39.6°, whereas Ti_0.8_V_0.2_ exists as a shifted peak at 39.3° due to the lattice constant (a) values of 3.211 Å to 3.232 Å. The samples hot-forged at 700 °C, especially at low strain rates, demonstrated the (110) β peak and (200) and (220) diffraction peaks around 85°, indicating a greater presence or precipitation of the β phase than the samples heated to higher temperatures. This is consistent with the findings in additive manufacturing, where cooling of the material at a rapid rate from the β-field usually results in the formation of the martensitic α′ phase, whereas strain at a lower rate results in α + β phases [59,60]. Also, increasing the processing temperature to 900 °C, the obtained diffractograms demonstrate a reduction in the β phase and lead to a stabilized lamellar α-morphology, demonstrated by the stable intensity of the hexagonal (101) and (110) peaks [60]. The asymmetric broadening of the (101) α peak at 2θ ≈ 40.7° is an indication of the presence of β-Ti with a relatively low volume fraction of 2–6%, depending on the manufacturing approach of the alloy used, such as selective laser melting (SLM) and electron beam melting (EBM) [60]. The presence of the Al_6_Ti_19_ phase, identified by hcp reflections, also reveals a complicated intermetallic substructure in the primary α-grains [59].

The dislocation density of the base Ti64 alloy and hot-forged samples was evaluated using Scherer′s equation, as given in Equations (2) and (3) [61]. The θ and β (FWHM) values were obtained from the XRD graphs using Origin Pro software 2021. Based on these calculations, the dislocation density for the base alloy sample was determined to be 8.84 × 10^−16^ mm^−2^, respectively.

(2) D = K × λ × [β × cos(θ)]^−1^

where D represents the crystallite size, K is the Scherer constant, λ denotes the X-ray wavelength, β corresponds to the full width at half maximum (FWHM), and θ is the peak angle.

(3) δ = 1/D^2^

where δ represents the dislocation density. The dislocation density development was significantly influenced by the interrelation of the deformation extent and the deformation temperature, as summarized in Table 2.

The dislocation density increased from 5.84 × 10^−16^ mm^−2^ at low deformation extent to 2.53 × 10^−11^ mm^−2^ at moderate deformation extent, showing that dislocation proliferation and strain hardening prevailed at the moderate strain extent. However, with an increase in deformation beyond the moderate extent, the dislocation density dropped to 8.17 × 10^−16^ mm^−2^, showing that the thermally activated recovery processes were sufficiently effective to annul the formed dislocations [62]. At 800 °C, both the low and moderate strains had similar low values of dislocation density of 5.57 × 10^−16^ mm^−2^ and 5.71 × 10^−16^ mm^−2^, respectively, which shows that the increase in deformation temperature led to dislocation rearrangement and annihilation even at moderate strains. Conversely, the high-strain condition led to a higher value of dislocation density of 2.38 × 10^−11^ mm^−2^, meaning that dislocation production was higher than recovery only at higher strains. The tendency of maximum dislocation density to appear at higher strain values is typical for hot deformation, where the process of defect storage is postponed due to dynamic recovery [63]. However, another pattern was found at 900 °C, while the dislocation density increased to 7.05 × 10^−12^ mm^−2^ when subjected to moderate deformation, it sharply decreased to 2.05 × 10^−14^ mm^−2^ in the presence of high deformation. It is evident that such a sharp decrease in the dislocation density at high levels of deformation was due to the highly effective DRV process and possibly DRX of the microstructure. The process of DRV consists of consuming highly deformed zones by newly emerged strain-free grains that leads to a significant decrease in the stored dislocation density. Thus, the dislocation density decreases, although the strain value is relatively higher due to the domination of softening mechanisms over strain hardening [64]. The obtained results show that the dynamics of the dislocation density changes depend on the competition between strain hardening that produces new dislocations and thermally activated DRV and DRX that reduce their statistical value. For a relatively low temperature of 700 °C, dislocation storage prevails under moderate plastic deformation, while for a higher temperature of 900 °C, the competition is shifted towards DRV and DRX processes. Consequently, severe plastic deformation at 900 °C does not enhance the dislocation density anymore but instead results in its destruction [62].

Analysis of the XRD results was applied based on peak position and width. It was carried out in order to analyze the effect of temperature and rate of cooling on the voltage of grating of Ti64 alloy. In this study, a Cu target was used at a 10–90° scan range and a wavelength of 1.5418 Å. The macrolattice strain was obtained by evaluating the relative variation Equation (4), in the interplanetary distance of the selected diffraction peaks of the α-Ti phase, taking an unprocessed Ti64 as the reference sample.

(4) $$\varepsilon_{macro}=\frac{d-d_{0}}{d_{0}}=\frac{Sin\theta_{0}}{Sin\theta }-1$$

where d_0_ and θ_0_ are the values for the unprocessed reference sample; d and θ are the values for the heat-processed sample. Microstrains were evaluated using the width of the diffraction peaks of the α-Ti phase by applying the Williamson–Hall method as written in Equation (5).

(5) $$\beta Cos\theta =\frac{k\lambda }{D}+4\varepsilon_{micro}Sin\theta$$

The slope of the straight line drawn by plotting βcosθ  against 4sinθ values gives the isotropic microstrain value. The Williamson–Hall method was formulated to distinguish between the two effects of crystallite size and lattice strain on peak broadening [65]. The modern technique for investigating Ti64 involves fitting multiple peaks of the α-Ti phase with the Gaussian–Lorentzian and employing the FWHM values in Williamson–Hall analysis [66].

As seen in Table 3, negative macrostrains were observed in most of the heat-treated samples. Negative strain means that the positions of the α-Ti reflections shifted towards larger 2θ values compared to the untreated sample, which resulted in a reduction in the distances between corresponding crystal planes. In the case of positive strain at 700 °C moderate and 900 °C moderate, there was an expansion of the α-Ti lattice. The maximum macro-strain was found to be −0.4493% for the 800 °C high sample. Next comes −0.3457% for the 800 °C slow sample, which means the high and slow strain rates, particularly at 800 °C, cause greater contraction in the α-Ti lattice spacing than the untreated sample. However, the macroscopic residual stress is represented by the lattice strain obtained from a single symmetric θ–2θ scan. The highest reliability value in terms of microstrain was calculated as 0.2288% in the 700 °C high sample. In this sample, the R^2^ value of 0.822 from the Williamson–Hall linear regression shows that the peak broadening exhibits a reasonable linear relationship with 4sinθ. Microstrain values of 0.2033% and 0.1819% were also obtained in the 700 °C moderate and 900 °C slow samples, respectively, and R^2^ values were found to be above 0.80 in both samples. The strain component in the peak broadening becomes more pronounced at high Bragg angles and forms the basis of the line broadening approach put forward by Williamson and Hall [65].

High microstrain identified under high-cooling conditions at 700 °C reveals a higher heterogeneity of elastic distortions in the crystal lattice. In two-phase alloys like Ti64, high values of the expansions could be connected to the misfit of elastic deformations of α and β phases, phase transitions, density of dislocations, distortions of the crystal lattice due to the presence of dissolved components, and thermal expansion. In the case of the Ti64 alloy literature, it has been revealed that different phases should be considered independently, and phase-dependent microstrains can be obtained from the peak profile analysis [66,67]. Among all samples tested, the 800 °C moderate sample demonstrates the highest R^2^ value for the Williamson–Hall graph, with R^2^ = 0.902. The microstrain in this sample was 0.1586%, which is relatively low. However, this microstrain appears to be better captured by the linear model than the other microstrain values. In the 800 °C high sample, although the macroscopic lattice strain reached its maximum, the microstrain yielded R^2^ = 0.414. It is thus evident that peak broadening in this sample is not due to microstrain and crystallite size effects alone. Although the microstrain of the 900 °C medium sample is calculated as 0.0295%, the regression coefficient (R^2^ = 0.026) is low. As far as the heating conditions are concerned, it has been observed that macrostrain and microstrain do not always show the same trend. The largest macro lattice contraction has been seen in the 800 °C high sample, while the largest microstrain has been measured in the 700 °C high sample. The reason behind this is that the two variables represent different physical properties. While macrostrain is the systematic average distance of lattices, microstrain is the distribution of local lattice distance. Thus, large macrostrains do not necessarily mean large microstrains [68,69].

## 4. Conclusions

This study investigates the effects of deformation temperature and strain rate on the development of microstructure, hardness, and dislocation density of Ti64 alloy during hot forging. The results demonstrate that the thermomechanical response of Ti64 is governed by a temperature- and strain-rate-dependent competition between deformation-induced strain hardening and thermally activated restoration mechanisms. At 700 °C, the alloy remained a lamellar α + β structure with pronounced work hardening, but raising the temperature up to 800 °C led to the formation of fragments of the lamellar structure and globularization. The observed morphological changes were associated with progressive lamellar fragmentation and spheroidization of the α phase, indicating the increasing contribution of dynamic restoration with increasing deformation temperature. DRV, DRX, and globularization dominated at 900 °C. The reduced dislocation density measured at 900 °C provides further evidence for the increased activity of recovery and recrystallization processes at this temperature. It was found through the measurement of hardness that higher hardness occurred in outer layers due to higher deformation, dislocations, and smaller grains. This spatial variation in hardness is therefore consistent with the observed deformation heterogeneity and the corresponding variation in defect density and microstructural refinement across the samples. Dislocation density depended on the deformation conditions, based on the XRD analysis. Maximum dislocation density was observed at moderate deformations at 700 °C and high deformations at 800 °C; however, it sharply decreased at 900 °C owing to the dominating DRV and DRX. The results suggest that an increase in deformation temperature leads to the shift in the dominating process from strain hardening to dynamic softening. Therefore, within the scope of the present microstructural and hardness-based investigation, the 900 °C condition at a relatively low strain rate exhibited the most favorable microstructural response, characterized by enhanced globularization, dynamic restoration, and reduced dislocation density.

## Figures and Tables

**Table 1:** Composition of the base Ti64 alloy sample.

Element	Ti	Al	V	C	N	O	H	Fe	Y	Other
wt.%	87.86	6.12	4.07	≤0.80	≤0.05	0.27	≤0.0125	≤0.30	≤0.005	≤0.40

**Table 2:** Dislocation density (D.D.) calculations for the reference specimen and hot-deformed specimens under different conditions of deformation (Def.) temperature and strain rate.

Temperature	Low Def. D.D. (mm^−2^)	Moderate Def. D.D. (mm^−2^)	High Def. D.D. (mm^−2^)	Metallurgical Kinetics
700 °C	5.84 × 10^−16^	253,000 × 10^−16^	8.17 × 10^−16^	The highest dislocation density was observed at the moderate deformation level, indicating pronounced dislocation accumulation under intermediate deformation conditions.
800 °C	5.57 × 10^−16^	5.71 × 10^−16^	238,000 × 10^−16^	The elevated temperature promoted DRV; however, substantial dislocation accumulation was observed only under the highest deformation level, where strain hardening exceeded the recovery rate.
900 °C	8.34 × 10^−16^	70,500 × 10^−16^	205 × 10^−16^	At the highest temperature, DRV and DRX became increasingly dominant. Consequently, the dislocation density decreased at the highest deformation level despite the larger imposed strain.

**Table 3:** The calculated values of macro and micro strains depending on production parameters.

Sample	Average Macro Strain (%)	Williamson–Hall Micro Strain (%)	R^2^
Ref. Ti64	0.0000	0.0177	0.016
700 °C Low	−0.0184	0.1702	0.782
700 °C Moderate	+0.0191	0.2033	0.818
700 °C High	−0.1139	**0.2288**	0.822
800 °C Low	−0.3457	0.1517	0.754
800 °C Moderate	−0.1744	0.1586	**0.902**
800 °C High	**−0.4493**	0.1050	0.414
900 °C Low	−0.1669	0.1819	0.823
900 °C Moderate	+0.1261	0.0295	0.026
900 °C High	−0.1800	0.1314	0.539

## Data Availability

The original contributions presented in this study are included in the article. Further inquiries can be directed to the corresponding author.
